# Two-component system RstAB promotes the pathogenicity of adherent-invasive *Escherichia coli* in response to acidic conditions within macrophages

**DOI:** 10.1080/19490976.2024.2356642

**Published:** 2024-05-20

**Authors:** Ting Yao, Xingmei Liu, Dan Li, Yu Huang, Wen Yang, Ruiying Liu, Qian Wang, Xueping Li, Jiarui Zhou, Chen Jin, Yutao Liu, Bin Yang, Yu Pang

**Affiliations:** a TEDA (Tianjin Economic-Technological Development Area) Institute of Biological Sciences and Biotechnology, Nankai University, Tianjin, China; b The Key Laboratory of Molecular Microbiology and Technology, TEDA Institute of Biological Sciences and Biotechnology, Ministry of Education, Nankai University, Tianjin, China

**Keywords:** Adherent-invasive *Escherichia coli* (AIEC), RstAB, macrophages, acid, csgD, *asr*, biofilm

## Abstract

Adherent-invasive *Escherichia coli* (AIEC) strain LF82, isolated from patients with Crohn’s disease, invades gut epithelial cells, and replicates in macrophages contributing to chronic inflammation. In this study, we found that RstAB contributing to the colonization of LF82 in a mouse model of chronic colitis by promoting bacterial replication in macrophages. By comparing the transcriptomes of *rstAB* mutant- and wild-type when infected macrophages, 83 significant differentially expressed genes in LF82 were identified. And we identified two possible RstA target genes (*csgD* and *asr*) among the differentially expressed genes. The electrophoretic mobility shift assay and quantitative real-time PCR confirmed that RstA binds to the promoters of *csgD* and *asr* and activates their expression. *csgD* deletion attenuated LF82 intracellular biofilm formation, and *asr* deletion reduced acid tolerance compared with the wild-type. Acidic pH was shown by quantitative real-time PCR to be the signal sensed by RstAB to activate the expression of *csgD* and *asr*. We uncovered a signal transduction pathway whereby LF82, in response to the acidic environment within macrophages, activates transcription of the *csgD* to promote biofilm formation, and activates transcription of the *asr* to promote acid tolerance, promoting its replication within macrophages and colonization of the intestine. This finding deepens our understanding of the LF82 replication regulation mechanism in macrophages and offers new perspectives for further studies on AIEC virulence mechanisms.

## Introduction

Crohn’s disease (CD) is a chronic inflammatory intestine disease. The prevalence of CD is 322 and 318 per million in Europe and North America, respectively, and the number of new cases is increasing dynamically in developing countries.^1–3^ There is no cure for CD; despite medical treatment, nearly half of the patients who require therapy develop refractory disease. CD is associated with characteristic changes in the intestinal microbiome, including expansion of adherent-invasive *Escherichia coli* (AIEC). Penetration of the epithelial barrier, survival and replication in macrophages are important steps in AIEC expansion in patients with CD. In macrophages, AIEC encounters acidic, oxidative, genotoxic, and proteotoxic stresses.^4,5^ Hence, it is important for AIEC to combat the harsh environment. According to current reports, the mechanism that promotes AIEC intracellular replication mainly consists of three aspects: damaging the host cell autophagy function,^6–13^ forming a replication niche by assembling biofilm-like communities to prevent clearing by autolysosome,^14–16^ and tolerating the acid attack of macrophages.^7,8^

The two-component system (TCS) is a key strategy for pathogens to adapt to their living environments and contributes to bacterial survival during infection. The TCS consists of two proteins, one being the histidine kinase (HK) responsible for detecting environmental changes and the other being the response regulator (RR) that activates or represses downstream genes in response to the signal received from HK. RstAB comprises RstA (HK) and RstB (RR). The functions of RstAB in pathogenic bacteria have been extensively studied. RstAB promotes motility in *Salmonella* typhimurium,^17^ *Photobacterium damselae* subsp. damselae^18^ and *Vibrio alginolyticus*,^19^ but represses motility in *Clostridioides difficile*.^20^ RstAB is essential for the adhesion of *V. alginolyticus* to mucus^19^ and *Edwardsiella ictaluri* ^21^ to catfish skin and the invasion of *Salmonella* typhimurium^17^ to HeLa cells. RstAB promotes antibiotic resistance in *Yersinia pseudotuberculosis* ^22^ and *P. damselae* subsp. damselae.^18^ RstAB is also important for the colonization in animal models in avian pathogenic *E. coli* (APEC) strain E058,^23^ *S. enterica*,^24^ *P. damselae* subsp. damselae,^18^ and *E. ictaluri* .^21^ However, it remains unclear whether RstAB contributes to AIEC virulence.

Replication in macrophages is essential for AIEC LF82 expansion in patients with CD.^25,26^ LF82 establishes a replication niche by assembling biofilm-like communities called intracellular biofilm communities (IBCs), which protect it from phagolysosomal attacks for long-term survival and replicate in macrophages.^8^ Curli fibers are the main extracellular matrix composed of IBCs.^27^ The structural components and assembly factors of the curli fibers are encoded by two operons: *csgBA* and *csgDEFG*. CsgD controls the transcription of both operons as a master regulator of curli fiber production.^28–30^ *csgD* is activated by three factors (OmpR, RstA, and IHF) and repressed by two factors (CpxR and H-NS) in *E. coli*.^29,30^ RstA regulates *csgD* expression by directly binding to the RstA-box on the promotor of *csgD*, and activating *csgD* only under acidic conditions in *E.coli* .^29^ Adaptation to an acidified macrophage environment is crucial for intracellular survival and replication of LF82.^31,32^ The acid shock protein Asr, encoded by the *asr* gene, strongly supports the growth of *E. coli* under acidic conditions.^33^ A previous study has shown that RstA is involved in *asr* activation, as *asr* expression is reduced when *rstA* is deleted.^34^ RstA also contributes to acid tolerance during the gut colonization in enterohemorrhagic *E.coli* (EHEC) O157:H7 by regulating *asr* expression.^35^ However, whether RstAB regulates *csgD* and *asr* in LF82 and whether it contributes to bacterial replication in macrophages is unclear.

In this study, we found that RstAB contributes to the virulence of LF82 by comparing the colonization of *rstAB* mutant and wild-type (WT) in a mouse model of chronic colitis. We found that RstAB contributes to LF82 virulence by promoting intracellular bacterial replication in macrophages. Hence, we investigated the mechanisms by which RstAB affects LF82 replication by comparing the transcriptomes of *rstAB* mutant and WT when infected macrophages. Differentially expressed genes (DEGs) in LF82 were identified and combined with the RstA-box motif on the promoters of DEGs to study the regulatory mechanism of RstAB. We found that RstAB directly activated intracellular biofilm community formation gene *csgD* and acid shock gene *asr* to promote LF82 replication in macrophages. Finally, we demonstrated that in response to acidic conditions within macrophages, RstAB activates *csgD* and *asr*, thereby contributing to LF82 pathogenesis. Our work contributes to the understanding of the regulatory function of RstAB in AIEC, particularly in relation to virulence, potentially influencing the future management and treatment of CD.

## Results

### RstAB promotes LF82 virulence in chronic colitis mice

**Figure 1.**
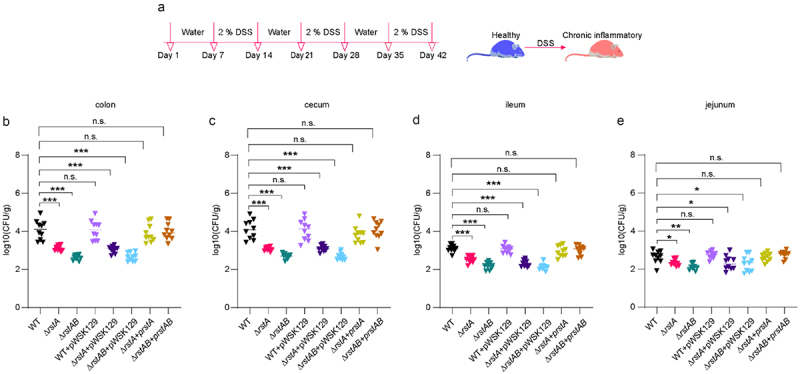
RstAB promotes LF82 virulence in chronic colitis mice.

To investigate whether RstAB contributes to LF82 virulence, a mouse model of dextran sulfate sodium (DSS) salt (w/v = 2%)-induced chronic colitis was established (Figure 1(a)). We recorded model data for chronic colitis; normally fed mice gained weight over time and stabilized after 35 days. DSS-treated mice lost 7.11% weight after three cycles of DSS administration, and the body weight recovered to a level similar to that of normally fed mice when DSS was replaced with water (Figure S1a). Intestinal edema in mice treated with DSS resulted in an increase in the weight of the cecum-connected colon to approximately 0.1 g (Figure S1b) and decreased the colon length to approximately 2 cm compared with that in normally fed mice (Figure S1c,d). The above characteristics exhibited the same trend as previously reported,^36^ indicating that the construction of a mouse model of chronic colitis was successful.

(a) DSS induced mouse model of chronic colitis.

(b-e) Bacterial titers of LF82 in mouse colon (b), cecum (c), ileum (d) and jejunum (e) infected with WT, Δ*rstA*, Δ*rstAB*, WT+pWSK129, Δ*rstA*+pWSK129, Δ*rstAB*+pWSK129, Δ*rstA*+p*rstA* and Δ*rstAB*+p*rstAB* at 24 h p.i. (*n* = 10).

Horizontal bars indicate the median. Data were obtained from three independent experiments and analyzed using the two-sided Mann – Whitney U test to calculate *P* values. *P< .05, **P < .01, *** *P* < .001; n.s., not significant.

Next, we constructed *rstA* and *rstAB* deletion mutants (Δ*rstA* and Δ*rstAB*), the complementary strains (Δ*rstA+*p*rstA* and Δ*rstAB+*p*rstAB*), and the control strains (WT+pWSK129, Δ*rstA*+pWSK129, and Δ*rstAB*+pWSK129). C57BL/6 mice were infected with Δ*rstA*, Δ*rstAB*, Δ*rstA+*p*rstA*, Δ*rstAB+*p*rstAB*, WT+pWSK129, Δ*rstA*+pWSK129, Δ*rstAB*+pWSK129, and WT, the bacterial titers in the mouse colon, cecum, ileum, and jejunum were calculated at 24 h post infection (p.i.). The bacterial titers of the colon, cecum, ileum, and jejunum were significantly lower of Δ*rstA* or Δ*rstAB* than that of WT in chronic colitis mice (Figure 1(b-e)). Δ*rstA*+p*rstA* and Δ*rstAB+*p*rstAB* restored the reduced bacterial titers of Δ*rstA* and Δ*rstAB* to a level that resembles WT in chronic colitis mice (Figure 1(b-e)). The bacterial titers of WT+pWSK129 colonizing the colon, cecum, ileum, and jejunum were not significantly different from those of WT mice (Figure 1(b-e)). Δ*rstA*+pWSK129 and Δ*rstAB*+pWSK129 failed to restore the colonization ability of Δ*rstA* and Δ*rstAB* in the colonized colon, cecum, ileum, and jejunum (Figure 1(b-e)), indicating that pWSK129 alone does not affect the colonization ability of LF82. This suggests that RstAB is essential for the colonization of LF82 in the intestine of mice with chronic colitis. Δ*rstA* and Δ*rstAB* grow as well as WT in LB and RPMI 1640 medium (Figure S2a,b), eliminating the possibility of the decreased LF82 colonization of Δ*rstA* or Δ*rstAB in vivo* to be due to a growth defect. These results suggest that RstAB is required by LF82 to effectively colonize in the intestine of mice with chronic colitis, thereby contributing to LF82 virulence.

### RstAB promotes the replication of LF82 in macrophages

**Figure 2.**
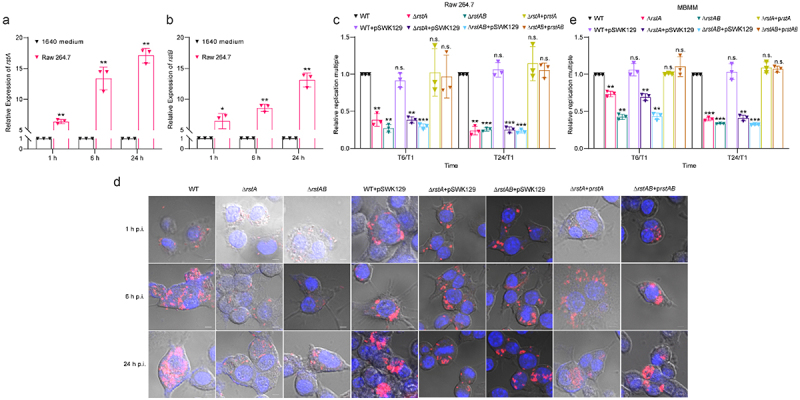
RstAB promotes the replication of LF82 in macrophages.

Macrophages in the mucous lamina propria of patients with CD are the main sites of LF82 replication, which is the basis for LF82 to invade deeper tissues across the intestinal barrier.^25^ Therefore, it is important to investigate the mechanisms underlying LF82 replication in macrophages. The expression of *rstA* and *rstB* was upregulated in LF82-infected Thp-1 or Raw 264.7 cells,^37^ suggesting that the expression of *rstA* and *rstB* is induced when LF82 survives in macrophages. Hence, we speculated that the decreased colonization of Δ*rstA* and Δ*rstAB* in chronic colitis mice is caused by reduced LF82 replication in macrophages. We investigated the expression pattern of *rstA* and *rstB* in LF82 when infected Raw 264.7 cells for 1, 6, and 24 h. The results showed that *rstA* was upregulated by 6.40-fold, 13.38-fold, and 17.09-fold, and *rstB* was upregulated by 6.40-fold, 8.56-fold, and 13.10-fold at 1, 6, and 24 h p.i. (Figure 2(a,b)), which is consistent with a previous study.^8^ These results suggest that the upregulation of *rstA* and *rstB* may help LF82 adapt to the intracellular environment of macrophages.

(a-b) qRT-PCR analyses the genes expression levels of *rstA* (a) and *rstB* (b) in LF82 cultured in RPMI 1640 medium or infected Raw 264.7 cells for 1, 6 and 24 h.

(c) Bacterial titers of WT, Δ*rstA*, Δ*rstAB*, WT+pWSK129, Δ*rstA*+pWSK129, Δ*rstAB*+pWSK129, Δ*rstA*-p*rstA* or Δ*rstAB*-p*rstAB* infected Raw 264.7 cells for 6 or 24 h relative to 1 h.

(d) Confocal microscopy determined the bacterial number of WT, Δ*rstA*, Δ*rstAB*, WT+pWSK129, Δ*rstA*+pWSK129, Δ*rstAB*+pWSK129, Δ*rstA*-p*rstA* or Δ*rstAB*-p*rstAB* in Raw 264.7 cells at 1, 6 or 24 h p.i.

(e) Bacterial titers of WT, Δ*rstA*, Δ*rstAB*, WT+pWSK129, Δ*rstA*+pWSK129, Δ*rstAB*+pWSK129, Δ*rstA*-p*rstA* and Δ*rstAB*-p*rstAB* infected MBMM for 6 or 24 h relative to 1 h.

The number of bacteria at 6 and 24 h p.i. was compared with that at 1 h p.i. by LB solid plate counting. The replication multiples at 6 and 24 h p.i. were expressed by T6/T1 and T24/T1, respectively.

Data were obtained from three independent experiments and analyzed using Student’s t-test. *P< .05; **P < .01; ***P < .001; n.s., not significant.

To investigate whether RstAB affects the replication of LF82 in macrophages, we infected Raw 264.7 cells with WT, Δ*rstA*, Δ*rstAB*, WT+pWSK129, Δ*rstA*+pWSK129, Δ*rstAB*+pWSK129, Δ*rstA+*p*rstA*, and Δ*rstAB+*p*rstAB* to assay the ability of intracellular replication at 6 and 24 h p.i. The replication of Δ*rstA* and Δ*rstAB* in Raw 264.7 cells were reduced by 1.4-fold and 2.3-fold at 6 h p.i. (Figure 2(c) and Table S1). The replication of Δ*rstA* and Δ*rstAB* was reduced by 2.5-fold and 3.0-fold compared with that of WT at 24 h p.i. (Figure 2(c) and Table S1). A more significant reduction of replication was obtained in Δ*rstA* and Δ*rstAB*-infected murine bone marrow-derived macrophages (MBMM) from chronic colitis mice. The replication of Δ*rstA* and Δ*rstAB* in MBMM was reduced by 2.6-fold and 3.7-fold at 6 h p.i. and reduced by 4.2-fold and 4.0-fold compared with that of WT at 24 h p.i., respectively (Figure 2(e) and Table S2). Δ*rstA+*p*rstA* and Δ*rstAB+*p*rstAB* restored the differences to similar levels of WT (Figure 2(c,e)). The ability of replication of WT+pWSK129 in Raw 264.7 cells and MBMM was not significantly different compared with that of WT (Figure 2(c,e)). Δ*rstA*+pWSK129 and Δ*rstAB*+pWSK129 failed to restore the replication ability of Δ*rstA* and Δ*rstAB* in Raw 264.7 cells and MBMM (Figure 2(c,e)), indicating that pWSK129 alone does not affect the replication of LF82 in macrophages. The confocal microscopy results showed the same trend as the intracellular replication results (Figure 2(d)). These results indicate that RstAB promotes the intracellular replication of LF82 in macrophages.

These results suggest that by promoting LF82 replication in macrophages, *rstAB* contributes to LF82 colonization in mice with chronic colitis, thereby enhancing LF82 virulence.

### RstAB promotes LF82’s replication in macrophages by regulating the expression of *csgD* and *asr*

**Figure 3.**
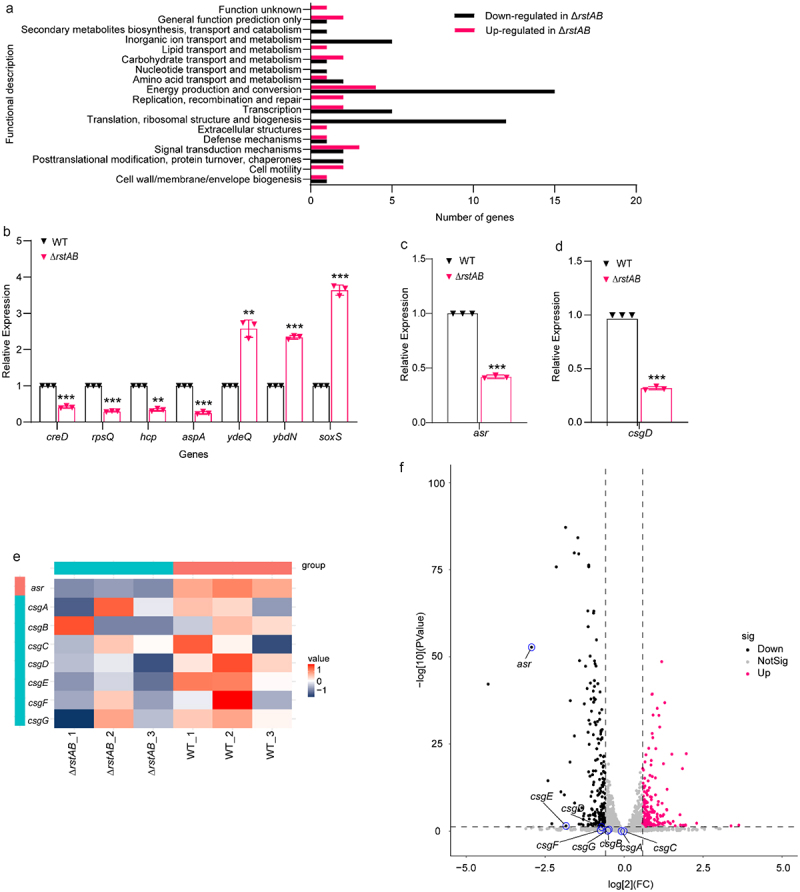
RstAB promotes LF82’s replication in macrophages by regulating the expression of *csgD* and *asr.*

To understand the downstream genes regulated by RstAB of LF82 in macrophages, the transcriptome of WT and Δ*rstAB* when infected Raw 264.7 cells for 1 h was performed by high-throughput Illumina RNA-sequencing (RNA-seq). The transcriptomic results showed that 83 genes were significantly differently expressed in the Δ*rstAB* group compared with that in the WT group, and 33 and 50 genes were categorized as up and downregulated in Δ*rstAB* (Table S3), respectively (fold change > 2 and *p* value < .05), indicating that RstAB acts as both an activator and a repressor in LF82 when infected macrophages. DEGs in the Δ*rstAB* compated with WT were classified using the NCBI Clusters of Orthologous Groups (COG) functional categories annotation system. The COG categories that were significantly enriched in the Δ*rstAB* group of upregulated genes were primarily associated with cell motility, signal transduction mechanisms, extracellular structures, replication, recombination, and repair. COG categories significantly enriched in the list of downregulated genes in the Δ*rstAB* group included post-translational modification, protein turnover, chaperones, translation, ribosomal structure and biogenesis, transcription, energy production, and conversion (Figure 3(a)). These results indicate that the impact of RstAB on LF82 gene expression is bidirectional, providing a basis for studying the mechanism of action of RstAB on the replication of LF82 in macrophages at the transcriptional level. We randomly selected seven DEGs to validate the result of the transcriptome by quantitative real-time PCR (qRT-PCR). The qRT-PCR results correlated well with the transcriptomic data, indicating that the transcriptome results were robust and valid (Figure 3(b)).

(a) COG analysis of DEGs in Δ*rstAB* when infected Raw 264.7 cells compared with that of WT at 1 h p.i.

(b) qRT-PCR analysis of the gene expression of DEGs in Δ*rstAB* when infected Raw 264.7 cells compared with that of WT at 1 h p.i.

(c) *asr* gene expression of WT and Δ*rstAB* when infected Raw 264.7 cells at 1 h p.i.

(d) *csgD* gene expression of WT and Δ*rstAB* when infected Raw 264.7 cells at 1 h p.i.

(e) Heatmap of *asr* and *csgABCDEFG* genes in Δ*rstAB* and WT when infected Raw 264.7 cells at1 h p.i.

(f) Volcano plot showing DEGs in the transcriptome of Δ*rstAB* when infected Raw 264.7 cells compared with that of WT at 1 h p.i.

Data were obtained from three independent experiments and analyzed using Student’s t-test. *P < .05, **P < .01, ***P < .001; n.s., not significant.

*csgD* and *asr* were found in the DEGs known to be regulated by RstAB in *E. coli* K12, with RstA-box sequences in their promoters,^21^ suggesting that RstA may bind to the promoters of *csgD* and *asr* in LF82. The expression of *csgD* and *asr* in WT and Δ*rstAB* when infected Raw 264.7 cells at 1 h p.i. were verified using qRT-PCR. The results showed that *csgD* and *asr* were downregulated in Δ*rstAB* when infected cells compared with WT (Figure 3(c,d)), which is consistent with the transcriptomic data (Figure 3(e,f)). We also verified the expression of *csg* gene cluster by qRT-PCR. Results showed that the expression of *csgA*, *csgB*, *csgC*, *csgE*, *csgF* and *csgG* was down-regulated in Δ*rstAB* when infected Raw 264.7 cells compared with that in WT (Figure S3). These results indicated that RstAB may positively regulate the expression of *csgD* and *asr* in LF82 upon infected macrophages.

### RstA regulates biofilm formation of LF82 by directly activates the expression of *csgD*

**Figure 4.**
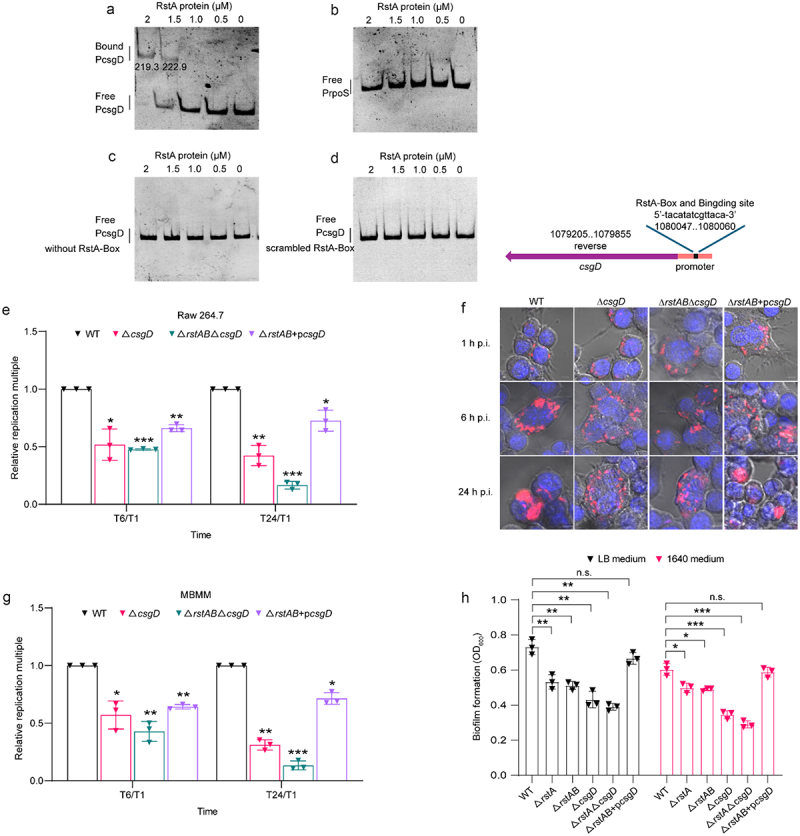
RstA regulates biofilm formation of LF82 by directly activates the expression of *csgD.*

*csgD* is a key transcriptional response regulator that controls curli fiber production and contributes to biofilm formation.^38^ As the RstA-box is present in the *csgD* gene promoter, an electrophoretic mobility shift assay (EMSA) was used to verify whether RstA directly binds to the *csgD* promoter. The results showed that with increasing concentrations of RstA protein, slowly migrating bands were observed for the *csgD* promoter region (gray value of bound DNA bands from left to right are 219.3, 222.9), but not *rpoS* (negative control) under the same conditions (Figure 4(a,b)). Deletion or scrambling of the RstA-box in the *csgD* promoter region completely abolished the binding of RstA to the *csgD* promoter (Figure 4(c,d)). These data suggest that RstA binds to the *csgD* promoter through the RstA-box to activate the expression of *csgD*.

(a-b) EMSAs of the binding of the promoter of *csgD* DNA fragment with purified RstA protein (a). *rpoS* promotor was used as the negative control (b). Images are representative of three independent experiments.

(c-d) EMSAs of the binding of purified RstA protein to the *csgD* promoter without RstA-box (c) or scrambled mutant of RstA-box (d). Images are representative of three independent experiments.

(e) Bacterial titers of WT, Δ*csgD*, Δ*rstAB*Δ*csgD* and Δ*rstAB*+p*csgD* in Raw 264.7 cells at 6 or 24 h relative to 1 h p.i. The replication multiples at 6 and 24 h p.i. were expressed by T6/T1 and T24/T1, respectively.

(f) Confocal microscopy determined bacterial number of WT, Δ*csgD*, Δ*rstAB*Δ*csgD* and Δ*rstAB*+p*csgD* in Raw 264.7 cells at 1, 6 or 24 h p.i.

(g) Bacterial titers of WT, Δ*csgD*, Δ*rstAB*Δ*csgD* and Δ*rstAB*+p*csgD* in MBMM at 6 or 24 h relative to 1 h p.i. The replication multiples at 6 and 24 h p.i. were expressed by T6/T1 and T24/T1, respectively.

(h) Biofilm formation of WT, Δ*rstA*, Δ*rstAB*, Δ*csgD*, Δ*rstAB*Δ*csgD* and Δ*rstAB*+p*csgD* in LB and RPMI 1640 medium.

Data were obtained from three independent experiments and analyzed using Student’s t-test. *P < .05, **P< .01, ***P < .001; n.s., not significant.

AIEC LF82 forms IBCs within phagolysosomes, which helps LF82 resist killing by phagolysosomes to survive, replicate, and provide a nutrient niche.^8^ Next, we investigated whether RstAB-activated *csgD* contributes to the intracellular replication of LF82 in macrophages. We constructed Δ*csgD*, *rstAB* and *csgD* double deletion (Δ*rstAB*Δ*csgD*), and Δ*rstAB*+p*csgD* mutants. Replication of Δ*csgD* in Raw 264.7 cells was 2.0-fold and 2.3-fold decreased, and Δ*rstAB*Δ*csgD* was 2.1-fold and 6.0-fold decreased compared with WT at 6 and 24 h p.i., and Δ*rstAB*+p*csgD* was 1.5-fold and 1.4-fold decreased compared with WT at 6 and 24 h p.i., respectively (Figure 4(e) and Table S4). In MBMM, the replication of Δ*csgD* was 1.8-fold and 3.2-fold decreased, and Δ*rstAB*Δ*csgD* was 2.3-fold and 7.5-fold compared with that of WT at 6 and 24 h p.i., and Δ*rstAB*+p*csgD* were 1.6-fold and 1.4-fold decreased compared with that of WT at 6 and 24 h p.i., respectively (Figure 4(g) and Table S5). The confocal microscopy results showed a consistent trend (Figure 4(f)). The reduced replication ability of Δ*csgD* to similar extent as that of Δ*rstAB*Δ*csgD* compared with WT at 6 h p.i. (Figure 4(e,g)). This difference was more pronounced in the Δ*rstAB*Δ*csgD* than Δ*csgD* at 24 h p.i. (Figure 4(e,g)). Additionally, the Δ*rstAB*+p*csgD* could not fully restore LF82‘s replication ability in macrophages (Figure 4(e,g)), suggesting that *csgD* is not the sole downstream virulence gene of RstAB. These results suggest that *csgD* affects intracellular replication and is regulated by RstAB; however, RstAB affects intracellular replication not only by regulating *csgD*.

CsgD is an important factor in the construction of an LF82-specific biofilm matrix and facilitates IBC formation within macrophages. To evaluate the effect of RstAB in biofilm formation by regulating *csgD* in LF82, a crystal violet staining assay was performed to quantify biofilm formation by WT, Δ*rstA*, Δ*rstAB*, Δ*csgD*, Δ*rstAB*Δ*csgD*, and Δ*rstAB*+p*csgD*. Biofilm formation was significantly decreased in Δ*rstA*, Δ*rstAB*, Δ*csgD*, and Δ*rstAB*Δ*csgD* compared with that of WT, and Δ*rstAB*+p*csgD* restored the biofilm formation ability of LF82 in LB and RPMI 1640 medium (Figure 4(h)). Moreover, the formation of biofilm of Δ*rstAB*Δ*csgD* was similar to that of Δ*csgD* (Figure 4(h)), indicating that RstAB influenced biofilm formation only through regulating *csgD*. These results suggest that RstA promotes LF82 biofilm formation by activating *csgD*.

These data indicate that RstA contributes to LF82 biofilm formation by directly activating *csgD* expression and promoting the replication of LF82 in macrophages.

### RstAB regulates the acid stress response of LF82 by directly activates the expression of *asr*

**Figure 5.**
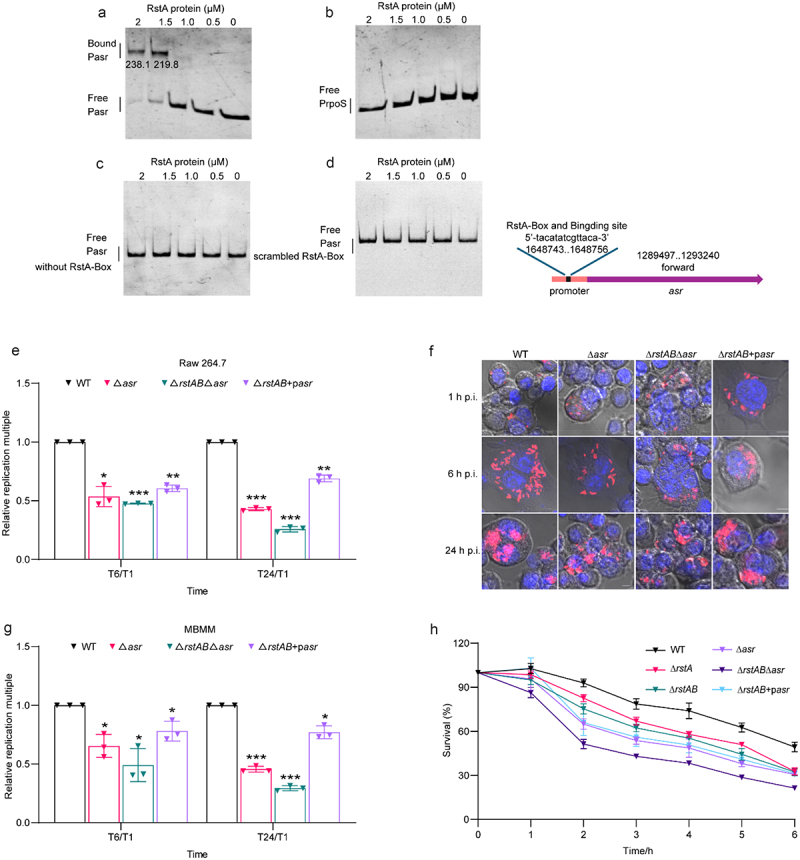
RstAB regulates the acid stress response of LF82 by directly activates the expression of *asr.*

Asr is an acid shock protein encoded by the *asr* gene. The EMSA results showed that with increasing concentrations of RstA protein, slowly migrating bands were observed for *asr* promoter (gray value of bound DNA bands from left to right are 238.1, 219.8), whereas no retarded bands were observed for the negative control (Figure 5(a,b)), indicating that RstA binds to *asr* promoter *in vitro*. Deletion or scrambling of the RstA-box in the *asr* promoter region completely abolished the binding of RstA to the *asr* promoter (Figure 5(c,d)). These data suggest that RstA binds to the *asr* promoter through the RstA-box to activate the expression of *asr*.

(a-b) EMSAs of the binding of the promoter of *asr* DNA fragment with purified RstA protein (a). *rpoS* promotor was used as the negative control (b). Images are representative of three independent experiments.

(c-d) EMSAs of the binding of purified RstA protein to the *asr* promoter without RstA-box (c) or scrambled mutant of RstA-box (d). Images are representative of three independent experiments.

(e) Bacterial titers of WT, Δ*asr*, Δ*rstAB*Δ*asr* and Δ*rstAB*+p*asr* in Raw 264.7 cells at 6 or 24 h relative to 1 h p.i. The replication multiples at 6 and 24 h p.i. were expressed by T6/T1 and T24/T1, respectively.

(f) Confocal microscopy determined bacterial number of WT, Δ*asr*, Δ*rstAB*Δ*asr* and Δ*rstAB*+p*asr* in Raw 264.7 cells at 1, 6 or 24 h p.i.

(g) Bacterial titers of WT, Δ*asr*, Δ*rstAB*Δ*asr* and Δ*rstAB*+p*asr* in MBMM at 6 or 24 h relative to 1 h p.i. The replication multiples at 6 and 24 h p.i. were expressed by T6/T1 and T24/T1, respectively.

(h) The tolerance of WT, Δ*rstA*, Δ*rstAB*, Δ*asr*, Δ*rstAB*Δ*asr* and Δ*rstAB*+p*asr* to low pH, determined by count of viable bacteria at indicated time points.

Data were obtained from three independent experiments and analyzed using Student’s t-test. *P < .05, **P < .01, ***P < .001; n.s., not significant.

The acidic environment in macrophages is required for the replication of LF82.^31^ Next, we constructed Δ*asr*, *rstAB* and *asr* double deletion strain (Δ*rstAB*Δ*asr*), and Δ*rstAB*+p*asr* mutants to investigate whether RstAB-activated *asr* contributes to intracellular replication of LF82 in macrophages. Replication of Δ*asr* in Raw 264.7 cells was 1.9-fold and 2.3-fold decreased, and Δ*rstAB*Δ*asr* was 2.1-fold and 3.8-fold compared with that of WT at 6 and 24 h p.i., and Δ*rstAB*+p*asr* was 1.7-fold and 1.5-fold decreased compared with that of WT at 6 and 24 h p.i., respectively (Figure 5(e) and Table S6). In MBMM, the replication of Δ*asr* was 1.5-fold and 2.2-fold decreased, and Δ*rstAB*Δ*asr* was 2.0-fold and 3.4-fold decreased compared with that of WT at 6 and 24 h p.i., and Δ*rstAB*+p*asr* was 1.3-fold and 1.3-fold decreased compared with that of WT at 6 and 24 h p.i., respectively (Figure 5(g) and Table S7). Confocal microscopy revealed a consistent trend (Figure 5(f)). These results indicate that RstAB-activated *asr* contributes to LF82 intracellular replication. The replication ability of Δ*asr* is weaker than that of WT (Figure 5(e,g)). This difference is more pronounced in the Δ*rstAB*Δ*asr* strain (Figure 5(e,g)). Additionally, the Δ*rstAB*+p*asr* could not fully restore LF82‘s replication ability in macrophages (Figure 5(e,g)), suggesting that *asr* is not the sole downstream virulence gene of RstAB. These results indicate that *asr* affects intracellular replication and is regulated by RstAB; however, RstAB affects intracellular replication not only by regulating *asr*.

To further investigate whether RstAB-activated *asr* helps LF82 in tolerating the acidic environment within macrophages, we conducted an acid tolerance assay. The tolerance of Δ*rstA*, Δ*rstAB*, Δ*rstAB*Δ*asr* and Δ*asr* to low pH decreased drastically within 2 h, and Δ*rstAB*Δ*asr* was weaker than Δ*asr*, Δ*rstAB*+p*asr* could not fully restore the reduced bacteria survival rate compared to WT (Figure 5(h)). These results indicated that RstA regulates *asr* to promote the survival of LF82 under acidic conditions, whereas RstAB promotes LF82 acidic tolerance not only by regulating *asr*.

### RstAB responds to acidic signal in macrophages and promotes intracellular replication of LF82

**Figure 6.**
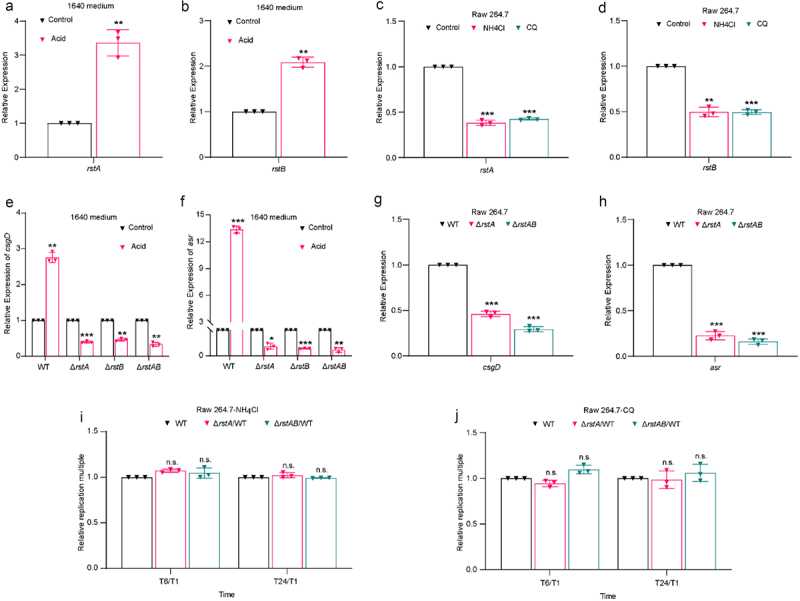
RstAB responds to acidic signal in macrophages and promotes intracellular replication of LF82.

In our previous study, we found that the expression of *rstA* and *rstB* was significantly upregulated in the transcriptome under acid treatment conditions, mimicking the acidic environment of macrophages, indicating that acid may activate the expression of *rstA* and *rstB*.^39^ qRT-PCR was performed to verify the transcriptome results. *rstA* and *rstB* were significantly upregulated by 3.4-fold and 2.1-fold in the acid-treated group compared with the control group, respectively (Figure 6(a,b)). Next, we investigated whether the acidic environment in macrophages regulates the expression of *rstA* and *rstB* by comparing the expression of *rstA* and *rstB* in LF82-infected normal and acid-neutralized macrophages. NH_4_Cl and chloroquine (CQ) were used to neutralize the acid within the macrophages.^31,32^ Acid neutralization with 30 mM NH_4_Cl or 10 μM CQ reduced the upregulated expression of *rstA* and *rstB* compared with the control group (Figure 6(c,d)), indicating RstAB response to acidic conditions in LF82-infected macrophages.

(a-b) qRT-PCR analyses the gene expression levels of *rstA* (a) and *rstB* (b) in WT cultured in acidic RPMI 1640 medium relative to neutral RPMI 1640 medium.

(c-d) qRT-PCR analyses the genes expression of *rstA* (c) and *rstB* (d) in WT infected Raw 264.7 cells treatment with NH_4_Cl or CQ relative to treatment without NH_4_Cl and CQ at 1 h p.i.

(e-f) qRT-PCR analyses the gene expression levels of *asr* (e) and *csgD* (f) in WT, Δ*rstA*, Δ*rstB* and Δ*rstAB* cultured in acidic RPMI 1640 medium relative to neutral RPMI 1640 medium.

(g-h) qRT-PCR analyses the genes expression of *asr* (g) and *csgD* (h) in Δ*rstA* or Δ*rstAB* relative to WT infected Raw 264.7 cells at 1 h p.i.

(i-j) Bacterial titers of WT, Δ*rstA* or Δ*rstAB* in Raw 264.7 cells treatment with NH_4_Cl (i) or CQ (j) relative to treatment without NH_4_Cl or CQ at 6 or 24 h relative to 1 h p.i.

The number of viable bacteria at 6 and 24 h p.i. was compared with that at 1 h p.i. The replication multiples at 6 and 24 h p.i. were expressed by T6/T1 and T24/T1, respectively.

Data were obtained from three independent experiments and analyzed using Student’s t-test. *P < .05, **P < .01, ***P < .001; n.s., not significant.

To determine whether RstAB regulates *csgD* and *asr* expression in response to acidic conditions, we assessed the mRNA levels of *csgD* and *asr* in LF82 cells cultured in acidic and neutral RPMI 1640 medium. qRT-PCR analysis showed that acid treatment increased the expression of *csgD* and *asr* in the WT (Figure 6(e,f)). However, *rstA, rstB*, and *rstA*B mutants abolished the upregulation of *csgD* and *asr* induced by acid (Figure 6(e,f)). These results indicated that acid is the signal that RstB senses to activate the transcription of *csgD* and *asr*. Moreover, the expression of *csgD* and *asr* in Δ*rstA* and Δ*rstA*B was also downregulated within Raw 264.7 cells compared with that of WT (Figure 6(g,h)), indicating that RstAB activates *csgD* and *asr* expression in LF82 within Raw 264.7 cells. These results suggest that, in response to acidic conditions in macrophages by RstB, LF82 RstAB actively regulates the expression of *csgD* and *asr*.

The acid-responsive TCS EvgAS activates PhoPQ via the small protein B1500, and PhoP activates transcription of *rstA* in a manner potentiated by EvgAS in *E. coli* .^40,41^ To investigate whether RstB directly or indirectly responds to acidic conditions, we constructed *evgAS* and *phoPQ* mutants (Δ*evgAS* and Δ*phoPQ*) and *evgASphoPQ* double mutant (Δ*evgAS*Δ*phoPQ*). Result showed that either Δ*evgAS*, Δ*phoPQ* mutant, or Δ*evgAS*Δ*phoPQ* reduces the up-regulation of *asr* and *csgD* under acidic condition (Figure S4A, B), indicating that acidic condition activates EvgAS or PhoPQ to regulate the *asr* and *csgD*. Combines with the above results, it suggests that in addition to RstB directly sensing the acidic conditions and regulating the expression of *asr* and *csgD*, EvgAS or PhoPQ also senses the acidic conditions and indirectly regulates the expression of *asr* and *csgD*.

We further verified whether acidic conditions in macrophages affect RstAB-dependent LF82 replication. Our previous study demonstrated that the neutralization of the acidic environment in macrophages with NH_4_Cl or CQ resulted in a reduction in LF82 replication by 3.3-fold and 4.2-fold, respectively.^39^ Whereas the replication ability of Δ*rstA* or Δ*rstAB* was unaffected in NH_4_Cl or CQ-treated Raw 264.7 cells compared with WT (Figure 6i,j). These results indicate that RstAB promotes the replication of LF82 in response to the acidic environment of macrophages.

## Discussion

RstAB is a critical TCS that plays a vital role in various pathogenic functions in bacteria. RstAB is a positive regulator of key virulence factors including damselysin, phobalysin P, and phobalysin C cytotoxins, in the marine pathogen *P. damselae* subsp. damselae.^18,42^ However, the signal by which RstAB responds to *P. damselae* subsp. damselae to affect virulence has not been clarified. RstA contributes to APEC E058 virulence by affecting chicken macrophage survival. RstA promotes APEC E058 to cause systemic infection in a chicken infection model.^23^ Deletion of *rstAB* increases the sensitivity of APEC to acidic conditions, as RstA positively regulates the acid resistance operons *hdeABD* and *gadABE* .^43^ RstA contributes to the bacterial colonization of EHEC O157:H7 i*n vivo*, by indirectly regulating LEE gene expression to promote bacterial adhesion *in vitro* and directly regulating the acid tolerance genes *asr* and *hdeA* .^35^ In this study, we found that RstAB promotes the replication of AIEC LF82 in macrophages, thereby contributing to its colonization in the intestine of mice with chronic colitis. Combined with studies of RstAB in various bacterial virulences, this indicates that RstAB is an essential virulence-regulatory TCS that responds to environmental cues.

Biofilms are bacterial aggregates embedded in a matrix of exopolysaccharides, DNA, and proteins that can shield bacteria from the immune system and antibiotics,^44,45^ leading to persistent and recurrent clinical infections.^46^ RstA negatively regulates biofilm formation in EHEC O157: H7 by controlling c-di-GMP biosynthesis.^35^ RstA also inhibits biofilm formation by *S. enterica* Typhimurium.^24^ In *Acinetobacter baumannii*, the RstA homology protein BfmR contributes to biofilm formation by activating the expression of the Csu pili chaperone – usher assembly system.^47^ Our results demonstrate that RstA contributes to biofilm formation in AIEC LF82 by directly activating *csgD* expression. These studies suggest that RstA either positively or negatively regulates biofilm formation in different pathogenic bacteria.^35,48,49^

Biofilm formation is a novel pathogenic feature of AIEC in the mucosa of patients with CD.^8^ AIEC infection causes intestinal dysbiosis by altering the microbiome, forming thick biofilms on the epithelium,^50^ and assembling IBCs within host cells to protect from phagolysosomal attack.^8^ Intracellular LF82 produces an extra-bacterial matrix that acts as a biofilm to control IBC formation.^8^ IBC also forms in the uropathogenic *E. coli* (UPEC)-infected bladder epithelial cell cytosol.^51^ The formation of IBCs provides a replication site for bacteria and contributes to recurrent urinary tract infections.^52^ AIEC and UPEC belong to the phylogenetic group B2,^53^ but the sites of IBC formation are different. UPEC forms IBCs in the cytosol of infected bladder epithelial cells, and AIEC forms IBCs inside phagolysosome.^54^ These results indicate that IBC formation is a crucial step in the intracellular pathogen life cycle. CsgD is the main regulator of curli fiber formation. Curli fibers are the main extracellular matrix that compose biofilms.^55^ The matrix of UPEC IBC contained curli fibers; hence, we speculated that curli fibers may also contribute to AIEC IBC formation. We found that RstAB contributes to LF82 biofilm formation by directly activating *csgD* expression and promoting the replication of LF82 in macrophages. These findings suggest that curli fibers are crucial for bacterial biofilm formation inside host cells.

AIEC survives in mature phagolysosomes and proliferates in low pH and protease environment.^31,32,56^ *S. enterica* Typhimurium is an intracellular pathogen that survives within *Salmonella*-containing vacuoles (SCVs), and the acidic conditions in SCVs is the signal for the expression of the pathogenicity island 2 type III secretion system and its secreted effectors.^57–59^ RstA contributes to *Salmonella* virulence by activating the expression of STM1485 (the homolog of *asr*).^33,60^ STM1485 is required to prevent the degradation of *Salmonella* in SCV and facilitate type III secretion system assembly, promoting intracellular bacterial replication in human epithelial cells and murine macrophages, thus affecting *Salmonella* virulence.^61^ In this study, we found that the deletion of *rstAB* led to the downregulation of *asr* gene expression in macrophages and that the deletion of *asr* gene weakened the acid tolerance and replication ability of LF82 in macrophages. These studies indicate that tolerance to the acidic environment within host cell vacuoles is a common strategy for the survival and replication of intracellular pathogenic bacteria.

We demonstrated that RstAB promoted the replication of AIEC LF82 in macrophages by enhancing LF82 biofilm formation and acid tolerance, which are necessary for its virulence of LF82. The replication results of single mutants (Δ*csgD* and Δ*asr*) and triple mutants (Δ*rstAB*Δ*csgD* and Δ*rstAB*Δ*asr*) in RAW 264.7 cells and MBMM were at the same level at 6 h p.i., whereas the replication of triple mutants (Δ*rstAB*Δ*csgD* and Δ*rstAB*Δ*asr*) was attenuated compared with single mutants (Δ*csgD* and Δ*asr*) at 24 h p.i. These results indicate that RstA regulates downstream replication-associated genes and contributes to LF82 replication. To investigate whether there are other genes regulated by RstA, we complemented the Δ*rstAB* with the ectopic expression of *csgD* or *asr*. The replication results showed that neither *csgD* nor *asr* fully compensated for the decreased replication capacity of Δ*rstAB*. RstAB regulates genes other than *csgD* and *asr* in *E. coli* K-12,^34^ suggesting that RstA regulates different downstream genes in various regulation patterns. Whether RstAB regulates additional genes in AIEC LF82 requires further investigation.

## Materials and methods

### Strains and plasmids

The bacterial and plasmids used in this study are listed in Table S7. *E. coli* LF82 O83:H1 was used as the WT strain. Gene deletion mutants were generated using the λ Red recombinase system supported by the pSim17 plasmid (blasticidin-resistant) encoding three proteins (Exo, Beta, and Gam) that are required for homologous recombination.^62,63^ A WT strain harboring the pSim17 plasmid was established using electrotransformation. Then, DNA fragments composed sequentially (5′→3′) of an upstream 39–45 bp sequence of the target gene, the chloramphenicol resistance gene sequence, and the downstream 39–45 bp reverse complementary sequence of the target gene were amplified using PCR. The chloramphenicol-resistant pKD3 plasmid was used as the template for PCR, and primers were designed to cover 39–45 bp homologous arm sequences. Next, DNA fragments were introduced into competent bacterial cells via electrotransformation. Finally, the cells were cultured at 37°C for 2 h, and the suspensions of recovered bacteria were spread onto agar plates containing 25 μg/mL of chloramphenicol to obtain single mutant colonies. To validate the correctness of the mutation, the target mutation loci of single chloramphenicol-resistant colonies were PCR-amplified and preliminarily identified using agarose gel electrophoresis. Further identification was performed by Sanger sequencing. The Δ*rstA*Δ*rstB* (Δ*rstAB*) double mutant was established based on a chloramphenicol-resistant Δ*rstA* mutant by introducing a kanamycin-resistance gene (from pKD4 plasmid) to replace the *rstB* gene.

We used pWSK129 for *rstA* and *rstAB* complementation, and pTrc99A for *csgD* and *asr* complementation. To express and purify the RstA protein with a 6×His-tag, the *rstA* fusion gene was cloned into the pET-28a (+) between the *NdeI* and *XhoI* sites, and the constructed plasmid were transformed into *E. coli* BL21 (DE3). Plasmid pUC57 carrying red fluorescent protein (mCherry) was used for confocal microscopy. All the constructed plasmids and strains were validated using the methods described for the identification of mutant strains. The plasmids, strains, and primers used in this study are listed in Table S7 and Table S8.

Bacteria were cultured overnight at 37°C in LB medium. When necessary, appropriate antibiotics were added: ampicillin, 50 μg/mL; kanamycin, 50 μg/mL; chloramphenicol, 25 μg/mL; gentamicin, 20 μg/mL, or 100 μg/mL.

### RNA-seq

The LF82 was inoculated in LB medium at a ratio of 1:1000 for overnight culture (approximately 16 h), and the overnight bacteria were inoculated in LB medium at a ratio of 1:100 for 6 h and centrifuged at 5,500 × *g* for 5 min. The supernatant was discarded to collect the bacteria and washed three times with RPMI 1640 medium containing 10% fetal bovine serum (FBS), incubated at 37°C for 30 min. Raw 264.7 cells were infected with LF82 at a multiplicity of infection (MOI) of 100:1 for 20 min, and then extracellular bacteria were killed with 100 μg/mL gentamicin for 40 min. Samples were collected to extract total RNA.

Total RNA was isolated using the TRIzol reagent (Invitrogen) according to the manufacturer’s instructions. The concentration and purity of the extracted RNA were detected using a Nanodrop 2000, the integrity of the RNA was detected using agarose gel electrophoresis, and the RNA integrity number was detected using an Agilent 2100. To ensure that the single database construction can meet the requirements of total RNA amount of ≥0.5 μg, concentration ≥ 45 ng/μL, and OD_260/280_ between 1.8 and 2.2. For library preparation, we used an amount ≥5 µg, concentration ≥ 800 ng/μL, and OD _260/280_ between 1.8 and 2.2 of total RNA per sample. rRNA was depleted from the total RNA using a Ribo-off rRNA depletion kit (Vazyme, Nanjing, China), and libraries were constructed and analyzed by Majorbio, Inc. (Shanghai, China). The eukaryotic cell data in each sample were removed, and the results of prokaryotic sequencing were mapped to the LF82 reference genome to obtain gene annotation and expression results. DEGs in Δ*rstAB* mutant compared with WT invading Raw 264.7 cells were identified using the DESeq R package. The resulting *p* values were adjusted using the Benjamini – Hochberg test to control for the false discovery rate. Genes with an adjusted *p* < .05 were considered differentially expressed. All the significant DEGs are listed in Table S2.

### qRT-PCR

RNA samples were isolated using TRIzol (Invitrogen), reverse transcribed using a PrimeScript RT reagent kit (Takara, Shiga, Japan), and processed for qRT-PCR. qRT-PCR was performed using the Applied Biosystems 7500 Real-Time PCR System and SYBR Green PCR Master Mix (Applied Biosystems). The fold change in the expression of the target gene relative to that of the housekeeping gene (16s) was determined using the 2^−ΔΔCt^ method.^64^ At least three biological replicates were used for each qRT-PCR. All oligonucleotides used for qRT-PCR are listed in Table S3.

### Cell culture and macrophage replication

Raw 264.7 cells, a murine macrophage cell line, were cultured with RPMI 1640 medium (containing 10% FBS and 1% penicillin and streptomycin when necessary) at 37°C in 5% CO_2_. Mouse bone marrow macrophages (MBMM), derived from chronic colitis inflammatory mice. After the mice were killed, the tibia and leg bones were taken, and the bone marrow was rinsed with RPMI 1640 medium (10% FBS + 1% P/S +10 μg/mL and macrophage colony-stimulating factor), and the impurities were filtered through the 70 μm cell strainer (Corning). The cell concentration was set at 10^6^ CFU/mL, and the cells were spread in a 12-well cell culture, which was used for bacterial infection experiments.

Bacterial survival and replication were measured using a gentamicin protection assay. Before infection, the bacteria were washed with phosphate-buffered saline (PBS) and resuspended in RPMI 1640 medium, incubated at 37°C with shaking at 180 rpm for 30 min, infected with a MOI of 100, centrifuged at 1,000 × *g* for 10 min, incubated with 5% CO_2_ at 37°C for 10 min, and then extracellular bacteria were killed with 100 μg/mL gentamicin for 40 min (defined as T1). To determine the number of bacteria in the cells, PBS and 1 mL of 1% Triton X-100 were added to each well and incubated for 5 min to lyse eukaryotic cells. Triton X-100 at this concentration had no effect on bacterial viability for at least 30 min. The samples were diluted and spread on LB medium agar plates to determine the number of colony-forming units (CFU) recovered from the cracked monolayers. T6/T1 or T24/T1 is the replication multiple of gentamicin treatment for 6 or 24 h compared with gentamicin treatment for 1 h. Mutations (T6/T1)/WT (T6/T1) or (T24/T1)/WT (T24/T1) were defined as the relative replication multiples.

### Growth curve

To determine the growth of each strain, overnight cultures were washed three times with PBS and diluted (1:1000) in LB medium without antibiotics. A 200-μL aliquot was added to a 96-well flat-bottom microplate, 200 μL of LB medium was added as a negative control, and incubated at 37°C with shaking at 180 rpm for 24 h, as previously described.^35^ The absorbance was recorded at 600 nm. The experiments were independently performed three times.

### Confocal observation

After infection with bacteria containing the mCherry plasmid, the macrophages were washed with PBS and fixed with 4% paraformaldehyde for 10 min. Subsequently, cells were washed with PBS and permeabilized with 0.1% Triton X-100 for 5 min. After washing with PBS, anti-fluorescence attenuation tablets (including DAPI) and anti-fluorescence quenching sealing solution (Beyotime, Shanghai, China) were dropped (10 μL at a ratio of 1:1) on the glass slide. The coverslips were mounted on slides, and cell images were acquired using a confocal laser scanning microscope (Zeiss LSM800) and analyzed with ZEN 2.3 (blue edition) and format designed using Adobe Illustrator CC 2018 software. The data were based on the clearest results preserved from five fields of three slides.

### Animal experiment

#### Mice

Six-week-old C57BL/6 L female mice purchased from Beijing Vital River Laboratory Animal Technology Co., Ltd. (Beijing, China) were housed under standard laboratory conditions (22 ± 1°C, 12:12-h light/dark cycle). All experiments were conducted according to protocols approved by the Institutional Animal Care Committee of Nankai University (Tianjin, China). All the mice were provided ad libitum access to a normal chow diet and water throughout the study.

### DSS-induced chronic colitis mouse model

C57BL/6 L female mice were modeled with reference to the animal model described by Kwon J et al..^36^ Chronic colitis was induced by oral administration of DSS (MW 36–50 kDa; MP Biomedicals, Santa Ana, CA, USA). After acclimation, to induce chronic colitis, 8-week-old mice were treated with three cycles of 2% (w/v) DSS for seven days, with seven days of drinking water between each cycle. After the 3rd cycle of DSS treatment, the mice were sacrificed, and tissue samples were harvested as previously described.^65^

### Gut colonization assay

Bacteria were cultured overnight in LB medium at 37°C and 180 rpm, then 1:100 was transferred to fresh LB medium and cultured to OD_600_ = 1.0. Bacteria were collected through centrifugation at 5,500 rpm and washed three times with PBS. A bacterial suspension of 2 × 10^10^ CFU/mL was prepared, and the mutants were mixed with the WT strain at a ratio of 1:1, and then gavaged (100 μL/mice). The bacteria were allowed to colonize the mice for 24 h before being sacrificed. The intestinal tissues were collected, the intestinal contents were removed, 1 mL of PBS and grinding beads were added for crushing in a tissue crushing instrument, and the samples were serially diluted and coated on LB solid medium with the corresponding resistance to count the number of bacteria.

### Acid tolerance assays

Overnight cultured bacteria were washed three times with PBS and then diluted to a concentration of 10^6^ CFU/mL in 1640 medium acidified to pH 3.0 with HCl. The cultures were incubated at 37°C for 0 to 6 h with shaking at 180 rpm. A 100-μL aliquot was removed from the flask and suitable dilutions were plated on LB agar once every hour. The experiments were performed independently three times.

### Biofilm formation assays

Biofilm formation was quantified by crystal violet staining as previously described.^35^ Overnight cultured bacteria were diluted in fresh medium (1:100) and incubated in 96-well polystyrene microtiter plates at 37°C for 24 h. Loosely associated bacteria were removed by washing thrice with PBS, and the remaining bacteria were stained with 0.5% crystal violet for 5 min. The biofilm was then destained by adding 200 μL of 95% ethanol to each well and quantified using an enzyme-linked immunosorbent assay plate reader at 590 nm. Each experiment was performed at least three times.

### EMSA

The 6 × His-tagged RstA protein was expressed and purified in E. coli BL21 (DE3). Target DNA fragments were amplified using PCR and purified using a SPARKeasy Gel DNA Extraction Kit (Sparkjade; #AE0101-C). Purified PCR fragments (40 ng) were incubated at 25°C for 30 min with 6× His-tagged RstA protein at concentrations ranging from 0 to 2 µM in 20-μL reactions containing binding buffer (1 mM Tris – HCl [pH 7.5], 0.2 mM dithiothreitol, 5 mM MgCl_2_, 10 mM KCl, and 10% glycerol, 30 mM acetyl phosphate). The protein-DNA fragments were electrophoretically separated on a native polyacrylamide gel at 4°C and 80 V/cm. The gel was stained for 10 min in a solution of 0.1% GelRed (Biotium; #41000), and protein bands were visualized using ultraviolet transillumination. The gray value of DNA was analyzed using ImageJ (NIH, https://imagej.nih.gov, accessed on 14 December 2022).

### Statistical analysis

Statistical analyses were conducted using the GraphPad Prism software (v9.4.0; GraphPad Software, San Diego, CA, USA). The mean ± SD from three independent experiments is shown in the figures. Differences between two mean values were evaluated using a two-tailed Student’s t-test or a two-sided Mann – Whitney U test to calculate the *p* values. Statistical significance was set at *p* < .05.

## Supplementary Material

Supplemental Material

## Data Availability

RNA sequencing data generated in this study are available from the NCBI SRA database. Accession to cite SRA data: PRJNA984413 https://www.ncbi.nlm.nih.gov/bioproject/?term=PRJNA984413.
