# Supplementary material for: Two-component system RstAB promotes the pathogenicity of adherent-invasive Escherichia coli in response to acidic conditions within macrophages
Source: Gut Microbes. 2024 May 20;16(1):2356642. doi: 10.1080/19490976.2024.2356642 (PMC11135836; doi:10.1080/19490976.2024.2356642)
Supplement: Supplemental Material [file KGMI_A_2356642_SM7046.zip › Supplementary materials20240518.docx]

**Supplemental Materials for**

**RstAB is an important regulator of adherent-invasive *Escherichia coli* LF82**

Ting Yao^a,b, †^, Xingmei Liu ^a,b, †^, Dan Li ^a,b,†^, Yu Huang ^a,b^, Wen Yang ^a,b^, Ruiying Liu ^a,b^, Qian Wang ^a,b^, Xueping Li ^a,b^, Jiarui Zhou ^a,b^,Chen Jin ^a,b^, Yutao Liu ^a,b^, Bin Yang ^a,b*^, Yu Pang ^a,b*^

*^a^ TEDA Institute of Biological Sciences and Biotechnology, Nankai University, Tianjin 300457, China; ^b^ The Key Laboratory of Molecular Microbiology and Technology, TEDA Institute of Biological Sciences and Biotechnology, Nankai University, Ministry* *of Education, Tianjin 300457, China.*

† Ting Yao, Xingmei Liu and Dan Li contributed equally to this study.

*Correspondence: yangbin@nankai.edu.cn (Bin Yang) † pangyu@nankai.edu.cn (Yu Pang).

**Supplemental Figures**


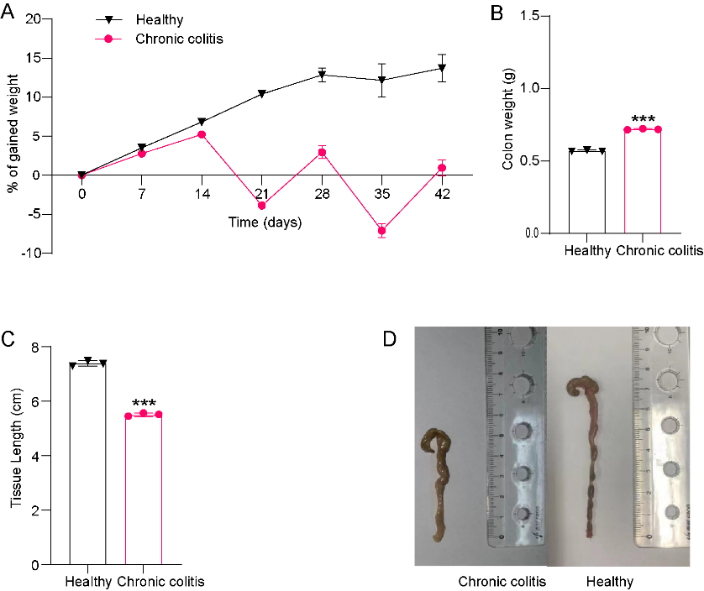


**Figure S1 Model index of chronic colitis induced by DSS**

Changes in (A) body weight, (B) colon weight, (C) colon length and (D) representative photographs for colon tissue of DSS-treated and non-treated mice. Data were obtained from three independent experiments and analyzed using Student’s t-test. *** *P* < 0.001; n.s., not significant.
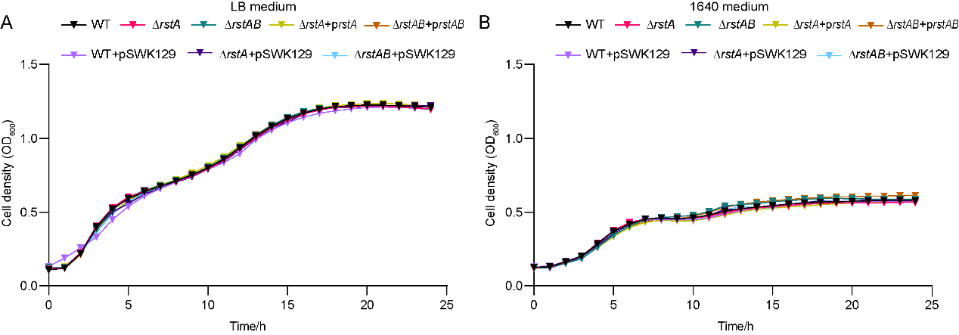


**Figure S2.** Growth curves of Δ*rstA*, Δ*rstAB*, Δ*rstA*-p*rstA*, Δ*rstAB*-p*rstAB*, WT+pWSK129, Δ*rstA*+pWSK129, Δ*rstAB*+pWSK129 and WT in LB medium (A) and RPMI 1640 medium (B). The absorbance of bacterial suspensions at 600 nm (OD_600_) was measured regularly using a microplate reader over a 24 hours time period. Data were obtained from three independent experiments and are presented as mean ± SD.


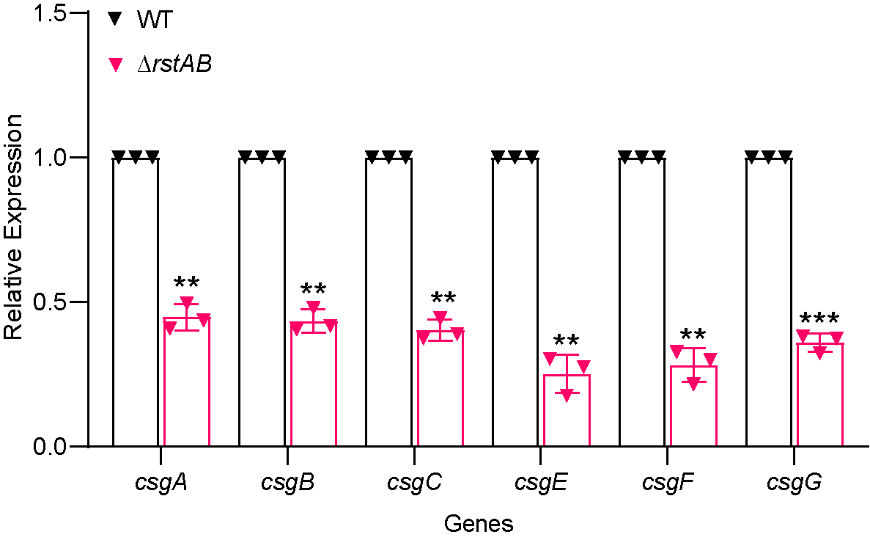


**Figure S3.** qRT-PCR analyses the genes expression levels of *csgA*, *csgB*, *csgC*, *csgE*, *csgF* and *csgG* genes expression of WT and Δ*rstAB*-infected Raw 264.7 cells at 1 h p.i.

Data were obtained from three independent experiments and analyzed using Student’s t-test. * *P* < 0.05, ** *P* < 0.01, *** *P* < 0.001; n.s., not significant.


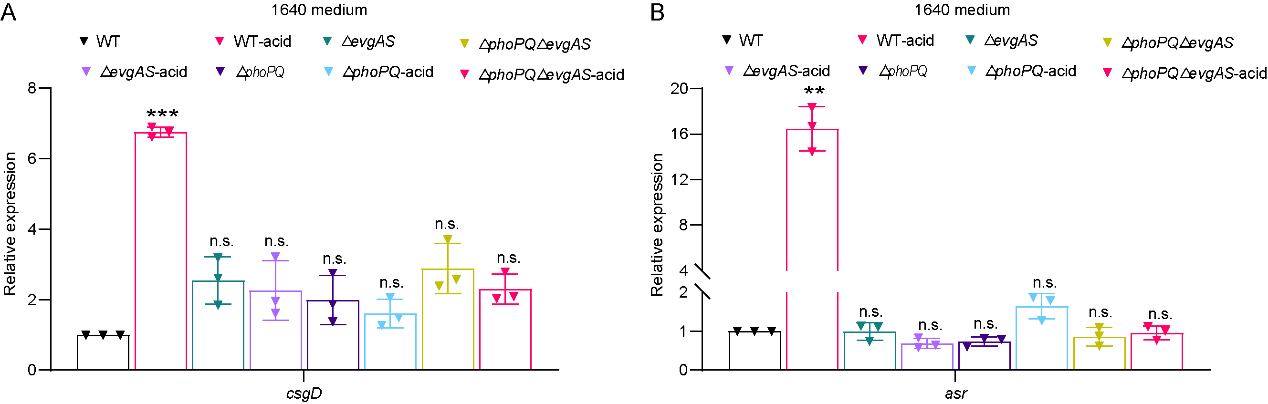
**Figure S4.** qRT-PCR analyses the genes expression levels of *csgD* and *asr* in WT, Δ*evgAS*, Δ*phoPQ* and Δ*evgAS*Δ*phoPQ* cultured in acidic RPMI 1640 medium relative to neutral RPMI 1640 medium.

Data were obtained from three independent experiments and analyzed using Student’s t-test. * *P* < 0.05, ** *P* < 0.01, *** *P* < 0.001; n.s., not significant.

**Table S1** The number of colony-forming unit of WT and *rstAB* mutants of LF82 at 1 h p.i., 6 h p.i. and 24 h p.i. within Raw 264.7 macrophages.

| Strains | 10^5^CFU/Well | | | | |
| --- | --- | --- | --- | --- | --- |
|  | T1 | T6 | T24 | T6/T1 | T24/T1 |
| WT | 3.12±0.21 | 5.60±1.11 | 48.67±11.02 | 1.81±0.46 | 15.64±3.80 |
| △*rstA* | 3.10±0.06 | 4.11±0.94 | 19.39±5.79 | 1.32±0.28 | 6.25±1.83 |
| △*rstAB* | 3.78±0.30 | 2.93±0.86 | 19.83±5.41 | 0.77±0.17 | 5.23±1.30 |
| WT+pWSK129 | 3.01±0.20 | 5.77±1.40 | 49.67±18.58 | 1.90±0.35 | 16.43±5.68 |
| △*rstA*+pWSK129 | 2.69±0.26 | 3.37±0.91 | 17.14±3.95 | 1.24±0.26 | 6.41±1.69 |
| △*rstAB*+pWSK129 | 3.29±0.15 | 2.61±0.86 | 16.48±3.91 | 0.79±0.23 | 5.01±1.15 |
| △*rstA*+p*rstA* | 3.50±0.07 | 6.40±1.61 | 59.33±11.93 | 1.83±0.47 | 16.93±3.06 |
| △*rstAB*+p*rstAB* | 3.03±0.19 | 5.97±1.17 | 50.67±13.32 | 1.97±0.35 | 16.62±3.37 |

**Table S2** The number of colony-forming unit of WT and *rstAB* mutants of LF82 at 1 h p.i., 6 h p.i. and 24 h p.i. within MBMM.

| Strains | 10^5^CFU/Well | | | | |
| --- | --- | --- | --- | --- | --- |
|  | T1 | T6 | T24 | T6/T1 | T24/T1 |
| WT | 8.65±0.49 | 22.67±3.82 | 79.17±6.29 | 2.62±0.44 | 9.15±0.30 |
| △*rstA* | 7.71±0.71 | 7.82±2.64 | 17.18±6.26 | 1.00±0.25 | 2.19±0.59 |
| △*rstAB* | 5.60±1.26 | 3.92±0.97 | 12.88±1.61 | 0.70±0.13 | 2.34±0.27 |
| WT+pWSK129 | 4.57±0.52 | 10.95±2.31 | 44.38±7.00 | 2.42±0.57 | 9.68±0.58 |
| △*rstA*+pWSK129 | 3.37±0.47 | 3.33±0.27 | 7.74±1.84 | 0.99±0.06 | 2.28±0.38 |
| △*rstAB*+pWSK129 | 4.12±0.46 | 3.14±0.39 | 8.53±0.13 | 0.77±0.10 | 2.09±0.25 |
| △*rstA*+p*rstA* | 4.06±0.60 | 10.69±2.99 | 42.14±7.65 | 2.71±0.97 | 10.50±2.38 |
| △*rstAB*+p*rstAB* | 4.51±0.54 | 11.21±2.79 | 43.45±6.51 | 2.54±0.83 | 9.63±0.88 |

**Table S4** The number of colony-forming unit of WT and *csgD* mutants of LF82 at 1 h p.i., 6 h p.i. and 24 h p.i. within Raw 264.7 macrophages.

| Strains | 10^5^CFU/Well | |  |  |  |
| --- | --- | --- | --- | --- | --- |
|  | T1 | T6 | T24 | T6/T1 | T24/T1 |
| WT | 5.18±0.33 | 12.97±1.66 | 70.90±14.50 | 2.49±0.17 | 13.64±2.45 |
| △*csgD* | 6.83±2.36 | 9.21±4.25 | 41.70±27.13 | 1.31±0.30 | 5.91±2.23 |
| △*rstAB*△*csgD* | 6.48±0.76 | 7.73±1.32 | 15.10±5.71 | 1.19±0.07 | 2.31±0.77 |
| △*rstAB*+p*csgD* | 5.15±0.73 | 8.49±1.31 | 50.49±9.88 | 1.65±0.04 | 9.75±0.56 |

**Table S5** The number of colony-forming unit of WT and *csgD* mutants of LF82 at 1 h p.i., 6 h p.i. and 24 h p.i. within MBMM.

| Strains | 10^5^CFU/Well | |  |  |  |
| --- | --- | --- | --- | --- | --- |
|  | T1 | T6 | T24 | T6/T1 | T24/T1 |
| WT | 5.83±1.32 | 13.89±3.20 | 59.95±16.41 | 2.39±0.30 | 10.36±1.96 |
| △*csgD* | 8.98±1.44 | 12.33±4.27 | 29.47±11.41 | 1.38±0.42 | 3.24±0.86 |
| △*rstAB*△*csgD* | 4.70±1.31 | 4.61±0.41 | 6.63±3.06 | 1.03±0.27 | 1.41±0.61 |
| △*rstAB*+p*csgD* | 4.18±0.89 | 6.37±1.17 | 30.57±7.24 | 1.54±0.2 | 7.33±0.92 |

**Table S6** The number of colony-forming unit of WT and *asr* mutants of LF82 at 1 h p.i., 6 h p.i. and 24 h p.i. within Raw 264.7 macrophages.

| Strains | 10^5^CFU/Well | |  |  |  |
| --- | --- | --- | --- | --- | --- |
|  | T1 | T6 | T24 | T6/T1 | T24/T1 |
| WT | 4.37±1.14 | 9.56±1.79 | 45.56±4.99 | 2.22±0.18 | 10.71±1.59 |
| △*asr* | 4.00±0.40 | 4.69±0.25 | 18.50±4.20 | 1.18±0.08 | 4.60±0.78 |
| △*rstAB*△*asr* | 5.68±0.77 | 6.00±0.99 | 15.57±2.68 | 1.06±0.09 | 2.74±0.24 |
| △*rstAB*+p*asr* | 5.93±1.12 | 7.91±1.22 | 43.04±3.68 | 1.34±0.14 | 7.37±0.94 |

**Table S7** The number of colony-forming unit of WT and *asr* mutants of LF82 at 1 h p.i., 6 h p.i. and 24 h p.i. within MBMM.

| Strains | 10^5^CFU/Well | |  |  |  |
| --- | --- | --- | --- | --- | --- |
|  | T1 | T6 | T24 | T6/T1 | T24/T1 |
| WT | 5.70±1.25 | 14.11±1.70 | 55.28±10.02 | 2.52±0.41 | 9.76±0.62 |
| △*asr* | 6.43±2.13 | 10.59±4.01 | 28.83±10.79 | 1.63±0.19 | 4.44±0.26 |
| △*rstAB△asr* | 6.11±1.74 | 7.81±3.82 | 17.56±5.47 | 1.22±0.32 | 2.86±0.10 |
| △*rstAB*+p*asr* | 5.21±0.98 | 10.72±4.99 | 39.13±8.45 | 1.99±0.55 | 7.51±0.54 |

**Table S8. Plasmids and strains used in this study.**

| **Plasmids** | | |
| --- | --- | --- |
| pKD3 | Carrying the chloramphenicol acetyltransferase gene | Lab collection |
| pKD4 | Carrying the kanamycin-resistant protein gene | Lab collection |
| pSim6 | Providing λ Red recombinase system | Lab collection |
| pWSK129 | pSC101-derivative plasmid with low copy number | Lab collection |
| pTRC99A | Carrying Trc promoter plasmid with low copy number | Lab collection |
| pET-28a (+) | Providing the DNA sequence of 6×His tags | Lab collection |
| pUC57 | Providing mCherry Fluorescent Protein | This study |
| pWSK129-*rstA* | pWSK129 carrying the DNA sequence of *rstA* gene and its promoter region | This study |
| pWSK129-*rstAB* | pWSK129 carrying the DNA sequence of *rstAB* | This study |
| **Strains** | **Genotype or description** | **Source** |
| BL21 | Expression strain | Lab collection |
| DH5α | *E. coli* DH5α/λpir strain | Lab collection |
| WT | Adherent-invasive *E. coli* O83:H1 strain LF82 | Lab collection |
| WT-mCherry | WT containing pUC57 | This study |
| WT- pWSK129 | WT containing pWSK129 | This study |
| WT- pWSK129-mCherry | WT containing pWSK129 *and* pUC57 | This study |
| Δ*rstA* | *rstA* deletion mutant in LF82 | This study |
| Δ*rstA*-mCherry | Δ*rstA* containing pUC57 | This study |
| Δ*rstA*+p*rstA* | *ΔrstA* containing pWSK129*-*p*rstA* | This study |
| Δ*rstA*+p*rstA*-mCherry | Δ*rstA*+p*rstA* containing pUC57 | This study |
| Δ*rstAB* | *rstAB* deletion mutant in LF82 | This study |
| Δ*rstAB*-mCherry | Δ*rstAB* containing pUC57 | This study |
| Δ*rstAB*+p*rstAB* | *ΔrstAB* containing pWSK129*-*p*rstAB* | This study |
| Δ*rstAB*+p*rstAB*-mCherry | Δ*rstAB*+p*rstAB* containing pWSK129*-*p*rstAB* and pUC57 | This study |
| Δ*csgD* | *csgD* deletion mutant in LF82 | This study |
| Δ*rstAB*Δ*csgD* | *csgD* deletion mutant in Δ*rstAB* | This study |
| Δ*csgD*-mCherry | Δ*csgD* containing pUC57 | This study |
| Δ*rstAB*Δ*csgD*-mCherry | Δ*rstAB*Δ*csgD* containing pUC57 | This study |
| Δ*asr* | *asr* deletion mutant in LF82 | This study |
| Δ*asr*-mCherry | Δ*asr* containing pUC57 | This study |
| Δ*rstAB*Δ*asr* | *asr* deletion mutant in Δ*rstAB* | This study |
| Δ*rstAB*Δ*asr*-mCherry | Δ*rstAB*Δ*asr* containing pUC57 | This study |
| Δ*evgAS* | *evgAS* deletion mutant in LF82 | This study |
| Δ*phoPQ* | *phoPQ* deletion mutant in LF82 | This study |
| Δ*evgAS*Δ*phoPQ* | *phoPQ* deletion mutant in Δ*evgAS* | This study |
| Δ*rstAB*+p*asr* | *ΔrstAB* containing pWSK129*-*p*asr* | This study |
| Δ*rstAB*+p*csgD* | *ΔrstAB* containing pWSK129*-*p*csgD* | This study |

**Table S9. Oligonucleotides used in this study (5`-3`)**

| **Gene** | **F/R** | **sequence** |
| --- | --- | --- |
| **Primers for gene mutation** | | |
| *rstA* | F | 5´ gcggtgtattgtgacgtttttatatctaccgtgaatgttGTGTAGGCTGGAGCTGCTTC 3´ |
| *rstA* | R | 5´ caataacaggtaaaactggataaacagttttttcatcgcATGGGAATTAGCCATGGTCC 3´ |
| *rstAB* | F | 5´ gcggtgtattgtgacgtttttatatctaccgtgaatgttGTGTAGGCTGGAGCTGCTTC 3´ |
| *rstAB* | R | 5´ tagcacaatggtggtgacttgaccaccgtgcgcatagtgATGGGAATTAGCCATGGTCC 3´ |
| *csgD* | F | 5´ tctgccgccacaatccagcgtaaataacgtttcatgtctATGGGAATTAGCCATGGTCC 3´ |
| *csgD* | R | 5´ tgtcaggtgtgcgatcaataaaaaaagcggggtttcatcGTGTAGGCTGGAGCTGCTTC 3´ |
| *asr* | F | 5´ cagtggggttaaatgaaaaaacaaattgagggtatgacaGTGTAGGCTGGAGCTGCTTC 3´ |
| *asr* | R | 5´ tcaggcgcgagggggcgcgccagcattactgttgaaaacATGGGAATTAGCCATGGTCC 3´ |
| *evgAS* | F | 5´ tacagggagaagggaaatgcttcattgcaaagggaataatctGTGTAGGCTGGAGCTGCTTC 3´ |
| *evgAS* | R | 5´ tggtaaatagctcccacatttgaacattgtgggagccgctatATGGGAATTAGCCATGGTCC 3´ |
| *phoPQ* | F | 5´ ttataacggatgcttaacgtaatgcgtgaagtatggacatatATGGGAATTAGCCATGGTCC 3´ |
| *phoPQ* | R | 5´ cgcgttacactattttaataattaagacagggagaaataaaaGTGTAGGCTGGAGCTGCTTC 3´ |
| **Primers for identifying the gene mutants** | | |
| *rstA* | F | 5´ ATAAGCGCAAAGGTCGGGAA 3´ |
| *rstA* | R | 5´ CCGCCATCTGGTTAAATGCG 3´ |
| *rstAB* | F | 5´ ATAAGCGCAAAGGTCGGGAA 3´ |
| *rstAB* | R | 5´ CTGCTGCGATTTTCGGACTG 3´ |
| *csgD* | F | 5´ CGCCACAATCCAGCGTAAAT 3´ |
| *csgD* | R | 5´ ACAGTCATTCTTCTTGCCCGT 3´ |
| *asr* | F | 5´ CCGGACTTATTGCCTGCTCA 3´ |
| *asr* | R | 5´ TGCACCCAACACTACAGCAA 3´ |
| *evgAS* | F | 5´ GCAAAATACTTTCTTCTGTCA 3´ |
| *evgAS* | R | 5´ GGATAAGCCGATTACGAT 3´ |
| *phoPQ* | F | 5´ CCCTGAATGATAAACACG 3´ |
| *phoPQ* | R | 5´ CTGAAATAAGCCTCAAAAC 3´ |
| **Primers for complement** | | |
| *rstA* | F | 5´ GGGGTACCttgcgcgtaaggcttcaatgccgc 3´ |
| *rstA* | R | 5´ GCTCTAGA ttattcccacgcatgaggcgcaaa 3´ |
| *rstAB* | R | 5´ GCTCTAGAtcaggcagaggtaaattgcgggat 3´ |
| *asr* | F | 5´ GGAATTCATGAAAAAACAAATTGAGGGTATG 3´ |
| *asr* | R | 5´ CGGGATCCTTACGCTGCGGGTTGTGCAGCAGG 3´ |
| *csgD* | F | 5´ GGAATTCATGTTTAATGAAGTCCATAGTATT 3´ |
| *csgD* | R | 5´ CGGGATCCTTATCGCCTGAGGTTATCGTTTGC 3´ |
| **Primers for qRT-PCR** | | |
| *rstA* | F | 5´ GCGTCAGAATGAGCAAGCC 3´ |
| *rstA* | R | 5´ GAGCGAGATTTCAGTGTTAGCC 3´ |
| *rstB* | F | 5´ TTGGCGTCGCATTTAACCA 3´ |
| *rstB* | R | 5´ CGAGTCGGGCATAAGTCAGC 3´ |
| *csgD* | F | 5´ GCGACATTGAAAACTGGCCT 3´ |
| *csgD* | R | 5´ TAAGGAGGGCTGATTCCGTG 3´ |
| *asr* | F | 5´ ATTAGCTCTGGTTGTTGCCG 3´ |
| *asr* | R | 5´ GGGGCTTTCTGTTCAGCTTTG 3´ |
| *csgA* | F | 5´ TCTGGCAGGTGTTGTTCC 3´ |
| *csgA* | R | 5´ CCGCCGTGCTGGGTAATG 3´ |
| *csgB* | F | 5´ GCAGGGAGGCTCAAAACT 3´ |
| *csgB* | R | 5´ TAAGCACCTTGCGAAATA 3´ |
| *csgC* | F | 5´ AAGTCAGACGAAGCAAGA 3´ |
| *csgC* | R | 5´ CAGTAACAACTATTTTCACCC 3´ |
| *csgE* | F | 5´ TAGTGATAAATGGGAAAGTG 3´ |
| *csgE* | R | 5´ CAGTGCAAAGACGACAGT 3´ |
| *csgF* | F | 5´ TTCCAGTTCCGTAATCCA 3´ |
| *csgF* | R | 5´ ACAGTAGCCCACCCAAAA 3´ |
| *csgG* | F | 5´ TAAAGAAGCCGCAAGACC 3´ |
| *csgG* | R | 5´ CGGAACAGCAGTGGAGAA 3´ |
| **Primers for verification of the RNA-seq results** | | |
| *creD* | F | 5´ GACGATAAAACCGTGGAGCG 3´ |
| *creD* | R | 5´ GCGAAACATCGAAATCGGCT 3´ |
| *rpsQ* | F | 5´ CTGCAAGGTCGCGTTGTTAG 3´ |
| *rpsQ* | R | 5´ CCGCATTCGTTGTTCTCGTC 3´ |
| *hcp* | F | 5´ TAAAATCATGGCGTGGCTGG 3´ |
| *hcp* | R | 5´ CCGTCGCTTTGACATTGACC 3´ |
| *aspA* | F | 5´ CGCAGCTATGGCAAACAAAGA 3´ |
| *aspA* | R | 5´ GACCGATATTGGCCAGCACT 3´ |
| *ydeQ* | F | 5´ TAATGTCGATGGCGGGTCAA 3´ |
| *ydeQ* | R | 5´ GATCAGTGTCGTACCAGCCG 3´ |
| *ybdN* | F | 5´ TCTGACAGCCGAAATTGCCC 3´ |
| *ybdN* | R | 5´ GGAGCGCAACCCAGTAAAAC 3´ |
| soxS | F | 5´ TTGACCAGCCGCTTAACATTG 3´ |
| soxS | R | 5´ ATCAAAAATCGGACGCTCGG 3´ |
| **Primers for EMSA** | | |
| *asr* | F1 | 5´ TGCGCAGTTATTCTACCGCT 3´ |
| *asr* | R1 | 5´ GGAATGGCAGACTTCCGTGA 3´ |
| *asr* | F2 | 5´GTTTGGCGCTATCGTTACACGCTG 3´ |
| *asr* | R2 | 5´CAGCGTGTAACGATAGCGCCAAAC 3´ |
| *csgD* | F1 | 5´ TGTGTGTAGTAATAAATCAGCCCT 3´ |
| *csgD* | R1 | 5´ ATGACGAAAGGACTACAGCGA 3´ |
| *csgD* | F2 | 5´ TAAACACACAACTAAATGCAACTC 3´ |
| *csgD* | R2 | 5´GAGTTGCATTTAGTTGTGTGTTTA 3´ |
| *r*poS | F | 5´ CAAGCAAAAGCCTGGTTCCG 3´ |
| *rpoS* | R | 5´ CCTACGCCCATAACGACACA 3´ |
| **Primers for RstA-purification** | | |
| *rstA* | F | 5´ GGAATTCCATATGATGAACACTATCGTATTTGTGGAA 3´ |
| *rstA* | R | 5´ CCGCTCGAGTTATTCCCACGCATGAGGCGCAAA 3´ |
| **Primers for WT identifying** | | |
| wzx | F | 5´ CGCTCTAATGAACTACTGTCTCC 3´ |
| wzx | R | 5´ TTTTCCGTGATTTCCCTTG 3´ |
| wzy | F | 5´ GTTTTAATCTGGTTTTCGCTACC 3´ |
| wzy | R | 5´ TGCTGTTTTACAAGTTTCTGCA 3´ |
